# Analytical method for simultaneous quantification of levodopa and carbidopa in the injectable oleogel formulation by HPLC

**DOI:** 10.1186/s13065-025-01410-8

**Published:** 2025-02-17

**Authors:** Deepa D. Nakmode, Souha H. Youssef, Soumyajit Das, Yunmei Song, Sanjay Garg

**Affiliations:** 1 Centre for Pharmaceutical Innovation, University of South Australia, North Terrace, Adelaide, SA 5000 Australia; 2 Department of Industrial and Molecular Pharmaceutics, Purdue University, West Lafayette, IN 47907 USA

**Keywords:** Sodium bisulfite, Drug release, Levodopa, Carbidopa, Green analytical chemistry

## Abstract

The developed method for simultaneous detection of levodopa and carbidopa was able to separate the peaks of the drug and sodium bisulfite in the in-vitro release samples and stability samples. Levodopa (LD), a pro-drug of dopamine, is used as the gold standard treatment for Parkinson’s disease. It is usually prescribed with carbidopa (CD) to prevent the conversion of levodopa to dopamine peripherally, thus reducing undesirable side effects. Both drugs are unstable at pH 7.4 beyond 24 h due to their oxidation, therefore 0.2% sodium bisulfite is added to the formulation as an antioxidant. The separation was performed by gradient elution using the Luna-C18 column (250 × 4.6 mm, 5 µm) at a flow rate of 1 ml/min. The mobile phase was composed of mobile phase A 30 mM potassium phosphate and acetonitrile (95:5, v/v) with 35 mM tetrabutylammonium hydrogen sulphate and mobile phase B containing 30 mM potassium phosphate and acetonitrile (50:50 v/v). Drug peaks were detected at 280 nm with retention times of 3.05 ± 0.001 min for LD and 3.64 ± 0.001 min for CD. The validation of the method according to US FDA guidelines and results were found to be within acceptable limits. The method was linear from 10–100 µg/ml (r^2^ = 0.999) and 10–100 µg/ml (r^2^ = 0.999) for LD and CD, respectively. The developed method was applied to studying the drug release from *in-situ* gel. The environmental impact of the developed method was evaluated using various greenness assessment tools.

**Graphical abstract:**

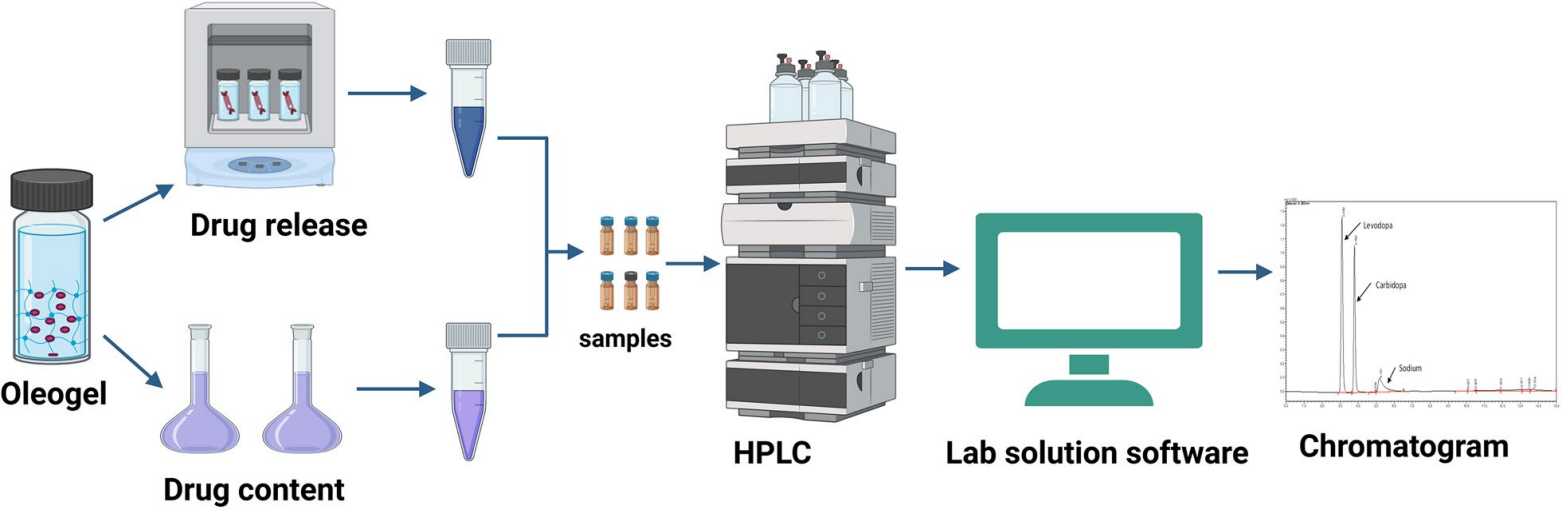

**Supplementary Information:**

The online version contains supplementary material available at 10.1186/s13065-025-01410-8.

## Introduction

Parkinson’s disease is a relentless neurological disorder, affecting more than 10 million people worldwide. As it progresses patients face immense challenges in movement and performing simple tasks. Dopamine replacement therapy is the main treatment protocol [1–3], where a combination of levodopa (LD) and carbidopa (CD) is used as the first-line therapy [4].

Patients are required to take oral tablets up to 4 times daily to maintain their normal day-to-day routine, posing inconvenience for patients and risking dosing errors such as accidental missed or repeated doses. The current treatment plan is necessary due to the short half-life and extensive metabolism of LD by the first-pass effect, leading to fluctuations in drug plasma concentrations [5].

**Fig. 1:**
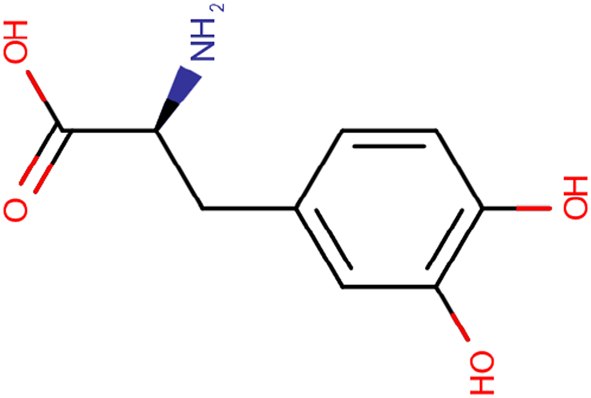
The chemical structure of Levodopa [8]

LD is a prodrug of dopamine, first introduced in 1967 by George Cotzias for the treatment of Parkinson’s [6]. Ever since LD (Fig. 1 The chemical structure of Levodopa) was considered the gold standard treatment for Parkinson’s, it has played a crucial role in combination therapy [7]. Following administration, LD quickly undergoes decarboxylation to dopamine in the plasma. The resulting dopamine then stimulates the peripheral dopamine receptors, which necessitates a higher dose of LD for achieving the therapeutic concentration in the central nervous system. The peripheral side effects of dopamine can further impact patients, including nausea and hypotension [7]. These side effects can be managed through the co-administration of 25% peripheral decarboxylase inhibitors such as carbidopa or benserazide.

### Carbidopa

**Fig. 2:**
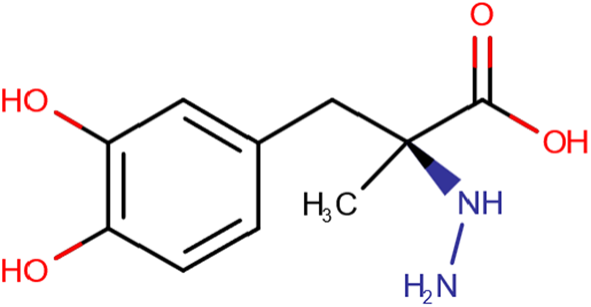
The chemical structure of carbidopa [11]

CD (Fig. 2 The chemical structure of carbidopa) is a competitive inhibitor of the enzyme aromatic l-amino decarboxylase, it cannot cross the Blood–brain barrier (BBB) and is mainly administered with LD for reducing the peripheral conversion of LD [9]. It reduces the systemic metabolism of LD and increases central exposure, which reduces the need for a high dose of LD to achieve the therapeutic concentration [10].

*In-vitro* release studies are an integral part of formulation development, allowing for optimization to achieve the targeted release profile. Both the drugs showed degradation in the alkaline pH (pH 7.4 phosphate buffer saline) as reported in the literature [12, 13].

Current treatment options include oral tablets, which require multiple time administration. The literature revealed some attempts to address the current limitations. For example, Sinemet CR developed by Merck Sharpe showed a more sustained plasma concentration of LD than the immediate release (IR) Sinemet tablet. However, LD bioavailability results for the Sinemet CR showed 71% bioavailability, whereas IR Sinemet showed 99% bioavailability [14]. Another formulation developed by Novartis Pharmaceutical, “Stalevo” tablet containing LD, CD, and entacapone for reducing the number of doses that need to be administered daily. Nevertheless, due to short duration of action of LD, this tablet needs to be administered every 3–4 h [15]. To bypass the oral route, an alternative approach was explored, where Duodopa, a continuous duodenal infusion, was developed by Abbvie Ltd. Duodopa offered continuous delivery of the LD and maintained constant plasma levels. Despite its advantages, duodopa is recommended for 16 h only. Furthermore, high probability of adverse events due to the device usage [5]. Consequently, improved formulations that can provide a sustained release of drugs are needed. The development of a biodegradable long-acting injectable formulation of LD and CD with a prolonged 7-day release was proposed in this study. The formulation of long-acting injectable formulation will reduce the dosing frequency, enhance patient compliance, improve bioavailability and bypass first-pass metabolism [16]. The injectable formulation will also maintain a constant plasma concentration of the drug which could reduce the levodopa-induced dyskinesia. The other marketed formulations include transdermal patches, extended-release pills, and dry powder inhaler however this formulations possess there drawbacks such as patch requires replacement every 24 h and dry powder inhaler causes cough [5].

Analysis is an essential component of product development, necessitating the accurate quantification of the formulated drugs. High-Performance Liquid Chromatography (HPLC) is the widely utilized technique in the analysis of active pharmaceuticals, impurities, and quality control of pharmaceutical products [17, 18], due to its reproducibility and accuracy. Several HPLC methods have been developed for LD and CD quantification as a single component as well as multicomponent mixtures with other drugs. The method reported by Wollmer et al. had a long run time (17 min) and high injection volume (30 µl) [19]. Raut et al. reported method used a high injection volume (50 µl) and involved a fluorescence detector which usually has limited availability in labs. In addition, this method involved a complex sample preparation procedure to impart fluorescence [20]. The technique developed by Kumar et al. utilized a high percentage of organic solvent 80% methanol [21]. Other methods involved the use of highly acidic mobile phases which can cause damage to the column [19]. The HPLC method established by Wikberg for the detection of LD, its metabolites, and CD, utilized a specialized electrochemical detector, which can be a regulatory bottleneck [22, 23]. Novel micellar HPLC–UV method have been developed by Belal and co-workers for the simultaneous detection of LD, CD and entacapone [24].

The literature revealed some other methods of analysis for the simultaneous determination of LD and CD including spectrophotometry [25], NMR spectroscopy [26], LC-EC [27, 28], LC–MS [29–31] and gas chromatography [32, 33]. However, reported methods had certain shortcomings, including a restricted linear range [34, 35], lack of data on selectivity and stability, and a deficiency in exploring the versatility of the analytical method [19, 34]. In addition, the published methods for simultaneous detection of LD and CD were not able to separate the peaks of the drug and sodium bisulfite in the in-vitro release samples and stability [19, 36, 37]. Hence, there is a need for a method that could separate all the peaks to enable the analysis of in-vitro release samples.

The use of organic solvent in the HPLC analysis contributes to environmental pollution when disposed improperly and poses serious health risks to the analysts [38]. Attempts to develop novel greener analysis procedures are continuously reported [38–40]. Although each analytical procedure by itself may not consume massive amounts of solvents and energy, the accumulation of the continuous process is not without environmental consequences. Scientists have been actively pursuing more environment friendly analytical procedures, especially after the introduction of the 12 principles of green analytical chemistry (GAC) in 1999 [41]. Publications discussing GAC have risen by more than 14-folds from 2001 to 2021 [42], demonstrating the increased awareness and the importance of assessing the possible environmental impact of newly developed methods. Therefore, the greenness evaluation of the developed HPLC method was performed using the analytical eco-scale assessment (ESA) [43], ComplexMoGAPI [44], and Analytical GREEnness Metric Approach and Software (AGREE) [45]. Additionally, Blue applicability grade index (BAGI) was explored to assess the applicability and convenience of the method [46].

## Experimental procedures

### Apparatus and software

Evaluation of LD and CD was performed using an HPLC system (Shimadzu Corporation, Kyoto, Japan) containing a binary pump (LC-20AD), a degasser (DGU-20A_3_), an autosampler (SIL-20A HT) and a single channel ultraviolet (UV) detector (SPD-20A). The stationary phase was the Luna-C18 column (250 × 4.6 mm, 5 µm) manufactured by Phenomenex. The software LabSolutions (version 5.92) manufactured by Shimadzu Corporation was used for processing and collecting data. A pH meter (Thermo Scientific, ORION STAR A111) was used for all pH measurements.

### Chemicals and solvents

LD and CD were purchased from Zhejiang Wild Wind Pharm (Zhejiang, China). White beeswax and Potassium dihydrogen orthophosphate anhydrous were purchased from Chemsupply (Gillman, Australia). Tetrabutylammonium hydrogen sulfate was purchased from Glentham Life Sciences (Corsharn, UK), and Sodium bisulfite, acetonitrile, and TRISMA base were purchased from Sigma-Aldrich (NSW, Australia). TRIZMA hydrochloride was purchased from Sigma-Aldrich (Steinheim, Germany).

### Chromatographic conditions

The analysis of samples was performed using a gradient elution program (Table 1). Mobile phase A was prepared by adding 0.5% triethylamine and 35 mM tetrabutylammonium hydrogen sulphate to 0.03 M potassium phosphate buffer (pH adjusted to 3.2 by phosphoric acid). The buffer was then mixed with acetonitrile at a ratio of 95:5, and v/v, respectively. Mobile phase B was similarly prepared using the 0.03 M potassium phosphate buffer and acetonitrile in the ratio 50:50, v/v. A flow rate of 1 ml/min was applied and a UV detector was utilized for detection. The signals were detected at 280 nm. The injection volume was 20 µl, and the column temperature was 40 °C.

**Table 1:** Optimized gradient profile

Time (min)	Mobile phase A (%)	Mobile phase B (%)
0	100	0
2	100	0
10	80	20
12	100	0
15	100	0

### Preparation of standard stock

10 mg LD and 10 mg CD were dissolved, separately, in 2 ml of 0.1 N phosphoric acid and the remaining volume was adjusted to 10 ml with mobile phase A achieving a final concentration of 1000 µg/ml for each drug. The prepared stock solution was stored at 4 °C and was used within a week.

Working concentrations were prepared by diluting the appropriate volume of standard stock with mobile phase A.

### Analytical method validation

#### Linearity and range

Accurately measured aliquots of LD and CD were transferred from the stock solutions and diluted using mobile phase A producing serial dilutions with concentrations range of 10–100 μg/ml for LD and CD. The samples were injected into the HPLC system, and the data was collected through LabSolutions software. A calibration curve was constructed by plotting the areas against their corresponding concentrations. Each data point represents the average of triplicate measurements.

#### Limit of detection (LOD) and limit of quantification (LOQ)

LOD and LOQ were calculated by using the following equations [47].

$$LOD=\frac{3.3*\sigma }{S},$$

$$LOQ=\frac{10*\sigma }{S},$$

where σ is the standard deviation (SD) of the response and S is the slope of the standard curve.

These values were obtained from the regression parameters generated from the standard calibration curve using GraphPad Prism (version 10.1.2) software.

#### Accuracy and precision

The solutions of three different concentrations of LD and CD representing low, medium, and high concentrations (25 μg/ml, 50 μg/ml, and 100 μg/ml) were prepared. Each sample was injected six times and Recovery percentages (R%) were calculated to assess accuracy. For precision, the same concentrations were analyzed at different times within the same day and on three different days to evaluate the intraday and interday precision, respectively.

#### Selectivity

For evaluating method selectivity, samples were prepared using release medium and mobile phase as diluents. The % recoveries (R%) and SD of the samples were calculated.

#### Robustness

Deliberate adjustments were applied to the developed chromatographic method conditions such as change in temperature (35 °C), molarity of buffer (35 mm), and the pH (3.4 buffer) of the mobile phase. Samples were analyzed under the modified conditions and their R% was calculated.

#### System suitability

The standard solutions of concentration 50 μg/ml for LD and CD were prepared for testing system suitability from stock solutions. Six replicate injections of the samples were injected. The system suitability parameters were evaluated according to the FDA guidelines [48].

#### Solution stability studies

Standard solutions of concentration 50 µg/ml of LD and CD were prepared, separately, using mobile phase and pH 7.4 buffer as diluents. The prepared samples were stored at room temperature and 4 °C for 14 days protected from light. The solutions were analyzed at predetermined intervals and recoveries were calculated.

### Oleogel development and application of developed method for oleogel studies

#### Preparation of oleogel

Miglyol 812, Beeswax, Tween 80, PEG 400, and sodium bisulfite were accurately weighed and placed in a stoppered glass tube and heated in a 100 ^o^C water bath. Once the ingredients melted, the tube was removed from the water bath and cooled to 40 ^o^C. CD and LD were then added to the mixture and mixed with a magnetic stirrer at 500 rpm for 1 h. The contents of the oleogel are summarized in Table 2. To avoid the peripheral conversion of levodopa to dopamine and associated side effects, carbidopa was also incorporated into the oleogel [9].

**Table 2:** Composition of levodopa and carbidopa loaded oleogel formulation

Component	Function	% w/w
Miglyol 812	Oily vehicle	75
Beeswax	Lipid/gelator	6
Tween 80	Surfactant	2
PEG 400	Vehicle	2.2
Sodium bisulfite	Antioxidant	0.22
Levodopa	Active ingredient	12
Carbidopa	Active ingredient	2.58

#### Determination of drug content

Accurately 15 mg of formulation was weighed in a 20 ml volumetric flask, and 1 ml of ethanol was added and vortexed to solubilize beeswax. The volume was made up to 20 ml with 0.1 N phosphoric acid and all the samples were sonicated for 30 min. 3 ml aliquots were withdrawn, filtered using a 0.22 µm polyvinylidene difluoride filter, and analyzed using the developed HPLC method. The same procedure was repeated for pure drugs and blank gel to validate the extraction method.

#### In-vitro release studies using the dialysis bag method

100 mg of the formulation was weighed in overnight soaked dialysis bags, which were tied at one end. Both ends of the bag were securely fastened with thread and bags were placed in glass vials with 10 ml Tris buffer containing 0.2% tween 80. The vials were then placed in an incubator at 37 °C at 250 rpm. Release media was completely replaced with fresh media every 24 h. The collected release samples were stored at – 20 ^o^C until analysis by HPLC.

## Results and discussion

### Analytical method development

LD and CD were found to be unstable in the release media (phosphate buffer saline pH 7.4), therefore an antioxidant was required to prevent degradation in the release media and enable their analysis. After screening different antioxidants in pH 7.4 tris buffer, 0.2% sodium bisulfite was added to the formulation to maintain the stability of both drugs under the studied conditions. An HPLC method was developed for separating LD and CD from sodium bisulfite peaks from the release samples. The LD and CD were successfully separated and quantified by the developed method. The chromatographic conditions were optimized for achieving acceptable resolution of LD, CD, and sodium bisulfite.

Different gradient systems with acetonitrile without tetrabutylammonium hydrogen sulphate were attempted, which separated both drugs, but sodium bisulfite was eluted with levodopa (refer to supplementary Fig. 5). The peak tailing was observed which was reduced after the addition of triethylamine at 0.5% concentration. Due to the cationic nature of sodium bisulfite, it has a tendency to bind to LD and CD. Consequently, a cationic ion quencher is necessary to facilitate the elution of the drug peaks where tetrabutylammonium hydrogen sulphate (TBAS) was added to the mobile phase, acting as a cationic ion pair reagent. In the reverse phase systems, acidic samples such as sodium bisulfite form an electrically neutral ion pair with the TBAS and are retained in the column. The ion-pair reagents used in the mobile phase form an ion-pair with ionic samples, which increases the hydrophobicity of the ion-pair which results in the higher affinity for the reverse stationary phase and hence increases sample resolution [49]. The addition of tetrabutylammonium hydrogen sulphate to the mobile phase A achieved acceptable peak resolution and improved peak shapes (refer to supplementary Fig. 9).

### Validation parameters

The validation of the developed HPLC method was performed according to the FDA guidelines [48]. The evaluation of all the validation parameters such as system suitability, linearity, accuracy, precision, robustness, selectivity, and stability were performed, and results were compared to those of reference values.

#### Linearity and range

The calibration curves for both drugs exhibited linear behavior within the concentration ranges of 10–100 µg/ml, with regression coefficient (r) > 0.999. Details of the regression parameters are provided in Table 3.

**Table 3:** Regression parameters for the developed chromatographic method (n = 6)

Analyte	Standard curve	Analytical range (µg/ml)	Slope	y-intercept	Standard error of slope	Standard error of intercept	LOD (µg/ml)	LOQ (µg/ml)
LD	Day 1	10–100	15,205	− 25,098	119.10	6928	3.27	9.91
	Day 2	10–100	14,960	− 19,936	90.72	5277		
	Day 3	10–100	14,983	− 18,769	117.61	6838		
CD	Day 1	10–100	12,014	− 12,929	50.94	2963	1.59	4.82
	Day 2	10–100	11,818	− 9528.1	57.78	3361		
	Day 3	10–100	11,951	− 8559.7	88.58	5153		

#### LOD and LOQ

Based on regression parameters derived from GraphPad Prism (version 10.1.2), the σ value for LD was found to be 15,066 and 5788 for CD. By substituting in Eq. (2) stated in Sect. 3.4, the values of LOD and LOQ for LD were 3.7 µg/ml and 9.91 µg/ml, whereas LOD and LOQ for CD were 1.59 µg/ml and 4.82 µg/ml, respectively.

#### Accuracy

Accuracy was evaluated by calculating the R% of LD and CD at three concentration levels (low, medium, and high), the results were within the acceptable limits in the range of 98–102% [35] As summarized in Table 4.

**Table 4:** Accuracy of the developed HPLC method

Drug	Concentration (µg/ml)	R% ± SD (n = 6)
LD	25	101.00 ± 0.13
	50	98.90 ± 0.13
	100	100.77 ± 0.13
CD	25	99.89 ± 0.16
	50	98.46 ± 0.43
	100	101.00 ± 0.57

#### Precision

The intra-day and inter-day precision values of the established method was evaluated by measuring peak area and results were reported as % RSD (Table 5). The % RSD for both the drugs were found to be in acceptable limit of less 2%.

**Table 5:** Precision of the chromatographic method n = 3

Analyte	Concentration (µg/ml)	RSD %			
		Day 1		Day 2	Day 3
		Inter-day	Intra-day		
LD	25	0.114	0.334	0.160	0.698
	50	0.036	0.010	0.037	0.132
	100	0.025	0.029	0.155	0.009
CD	25	0.027	0.538	0.627	0.270
	50	0.159	0.770	0.136	1.023
	100	0.223	0.551	0.030	0.494

#### Selectivity

**Fig. 3:**
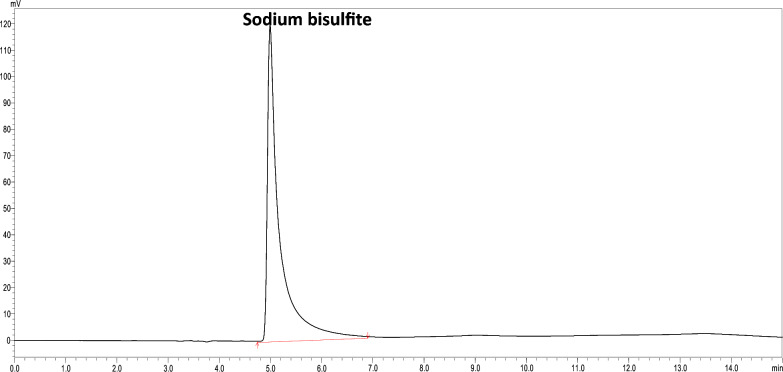
Chromatogram of the sodium bisulfite (R_t_: 4.99 min)

The samples prepared by diluting with release medium and mobile phase demonstrated acceptable recoveries (Table 6). No interference from the formulation excipients in the HPLC analysis of both drugs was observed. Also, no extra peaks were observed at a retention time of LD and CD in the blank gel samples. An additional peak was eluted at 4.99 min was sodium bisulfite peak shown in Fig. 3 from the release medium.

**Table 6:** Selectivity of the developed chromatographic method n = 3

Selectivity (R % ± SD)		
Sample	Release media (pH 7.4)	Mobile phase
LD	101.04 ± 0.04	100.86 ± 0.06
CD	101.72 ± 0.04	100.73 ± 0.17

##### Robustness

For evaluating the robustness of the chromatographic method intentional changes were introduced including pH change (+ 0.2), column temperature (− 5 °C) and change in molarity of potassium phosphate (35 mM). The slight changes in the chromatographic method did not affect the recoveries of the analyte as shown in the Table 7.

**Table 7:** Robustness of the chromatographic method

Parameters	R % ± SD	
	LD	CD
Temperature 35 °C	100.88 ± 0.01	101.05 ± 0.06
35 mM KH_2_PO_4_	102.01 ± 0.03	101.00 ± 0.83
pH of mobile phase (pH 3.4)	101.19 ± 0.48	98.73 ± 0.02

n = 3.

#### System suitability

System suitability parameters demonstrated results within acceptable limits specified by FDA guidelines (Table 8). Rs_1_ is the resolution between CD and LD, whereas Rs_2_ is between CD and sodium bisulfite.

**Table 8:** System suitability parameters of the developed HPLC method n = 6

Parameters		Analytes	
Name	Specification	LD	CD
Retention time (min)	–	3.05	3.64
Retention time (SD)	≤ 2	0.001	0.001
Plates	> 2000	34,128.67	45,813.00
HETP		29.30	21.83
Area (%RSD)	≤ 2	0.43	0.78
Tailing	≤ 2	1.31	1.23
Resolution (R_s1_)	> 2	–	3.2
Resolution (R_s2_)	> 2	–	3.3

#### Solution stability of the analytes

Both analytes were found to be stable in the mobile phase at room temperature (RT) and at 4 °C for up to 14 days, whereas stability samples in the release media (pH 7.4) demonstrated stability up to 14 days at room temperature as well as 4 °C, however, CD samples in release media at RT were only stable up to 3 days, beyond which CD began degrading (Table 9). These findings led to the conclusion that the release media needs to be replaced with fresh media every 24 h to allow for the quantification of the drugs in their intact form.

**Table 9:** Stability study results at different conditions n = 3

Drugs name	Testing conditions	R % ± SD			
		Day 1	Day 3	Day 7	Day 14
LD	Mobile phase at RT	100.06 ± 0.01	100.33 ± 0.04	100.04 ± 0.01	100.09 ± 0.02
	Mobile phase at 4 °C	100.02 ± 0.01	100.19 ± 0.05	100.01 ± 0.04	100.26 ± 0.06
	pH 7.4 at RT	99.71 ± 0.02	99.98 ± 0.00	99.82 ± 0.06	99.68 ± 0.02
	pH 7.4 at 4 °C	99.72 ± 0.02	99.88 ± 0.03	99.84 ± 0.05	99.53 ± 0.03
CD	Mobile phase at RT	99.49 ± 0.01	99.18 ± 0.02	98.90 ± 0.04	97.29 ± 0.05
	Mobile phase at 4 °C	99.76 ± 0.07	99.43 ± 0.09	99.76 ± 0.08	99.58 ± 0.23
	pH 7.4 at RT	98.11 ± 0.15	98.33 ± 0.09	96.69 ± 0.03	94.64 ± 0.07
	pH 7.4 at 4 °C	98.73 ± 0.05	98.47 ± 0.09	98.52 ± 0.12	98.41 ± 0.14

### Application of the developed analytical method in the oleogel formulation process

#### Drug content of the oleogel

**Fig. 4:**
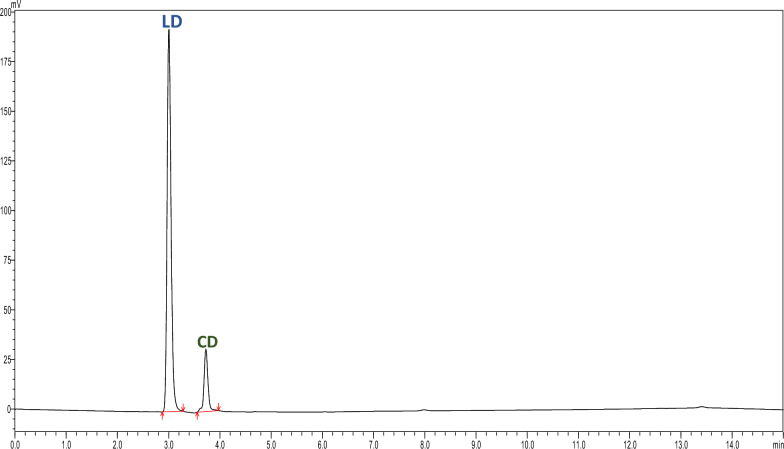
Chromatogram demonstrating separation Levodopa at 76.10 µg/ml (R_t_: 3.00 min) and Carbidopa at 15.10 µg/ml (R_t_: 3.72 min) from oleogel formulation

**Fig. 5:**
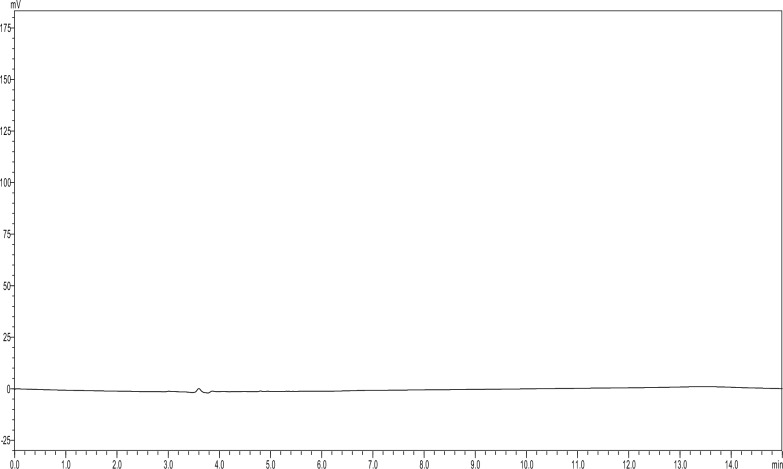
a Chromatogram of Blank oleogel formulation

**Fig. 6:**
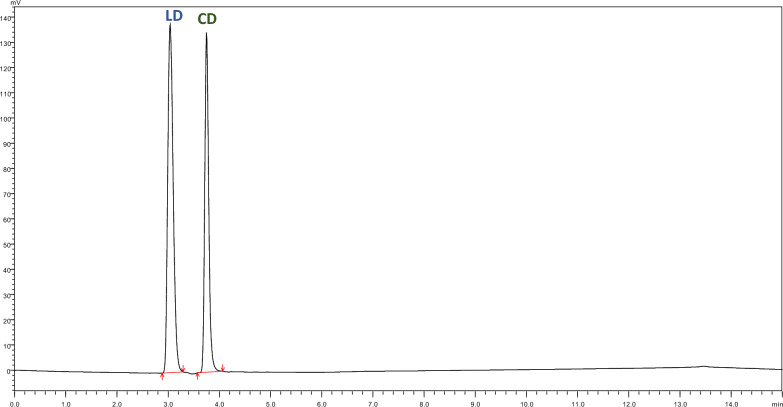
Chromatogram illustrating separation of the Pure drug mixture Levodopa at 60.53 µg/ml (R_t_: 3.03 min) and Carbidopa at 61.48 µg/ml (R_t_: 3.75 min)

The drug content of the prepared oleogel was successfully determined by the developed analytical method. The R% of LD and CD in the formulated oleogel was found to be 101.97% and 96.93% shown in Fig. 4, (Table 10) (the percentage of determined amount of the claimed amount). The same extraction method was used for the pure drugs for validating the extraction method, the R% for LD and CD was found to be 98.88% and 99.37%. No peaks were observed in the blank gel samples whereas the same extraction method was used for pure drugs no extra peaks were observed (Figs. 5 and 6).

**Table 10:** Application of the developed method for quantification of LD and CD in oleogel n = 3

Analyte	Drug name	Theoretical concentration (mg/ml)	Observed concentration (mg/ml)	Assay % ± SD
Formulation	LD	1.37	1.39	101.97 ± 4.76
	CD	0.340	1.32	96.93 ± 2.62
Pure drug	LD	0.245	0.242	98.88 ± 0.17
	CD	0.248	0.246	99.37 ± 1.80

#### Drug release study

**Fig. 7:**
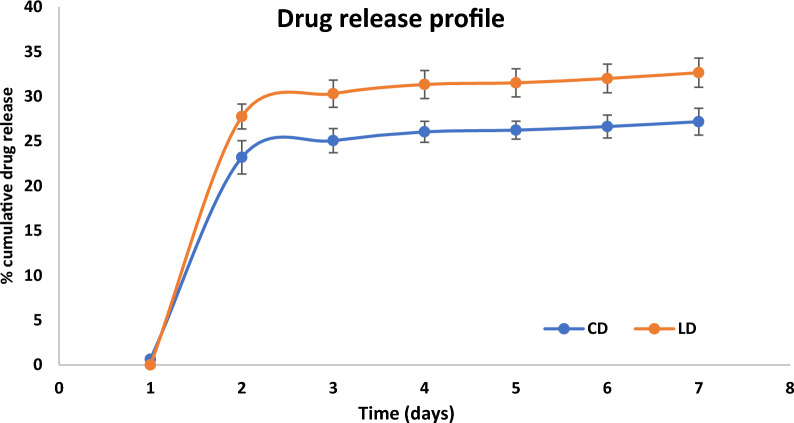
In-vitro drug release of LD and CD from Oleogel mean ± SE (n = 3)

**Fig. 8:**
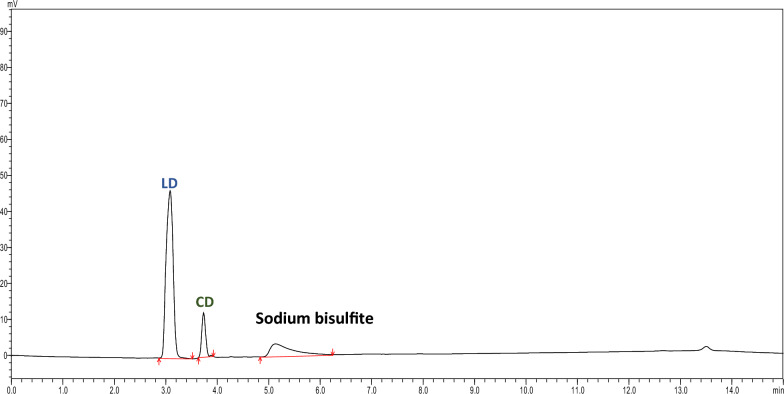
Chromatogram of Levodopa at 24.55 µg/ml (R_t_: 3.08 min) and Carbidopa at 4.76 µg/ml (R_t_: 3.73 min) from the release samples (Tris buffer pH 7.4)

The in-vitro drug release testing of the oleogel was performed using a dialysis bag. The developed analytical method was used for the simultaneous analysis of the *in-vitro* release samples. The cumulative drug (%) release was plotted against the corresponding time points (Fig. 7 In-vitro drug release of LD and CD from Oleogel). An initial burst release (27.75% for LD and 23.19% for CD) was observed followed by a secondary phase where the rate of release of LD and CD was slower release extending over 7 days. Figure 8 represents the chromatogram of the drugs after elution from the release medium, which shows the complete separation of both drugs from the sodium bisulfite peak.

## Evaluation of greenness of the chromatographic method

The proposed method was optimized with an environmentally aware mindset, utilizing minimal hazardous reagents, reducing waste generation, and lowering energy consumption wherever possible without compromising the quality of the results. The main solvents used in this study were ethanol for drug extraction from oleogels and acetonitrile, a component of the mobile phase. According to the GlaxoSmithKline solvent guide, their environmental impact was scored 8 (green shade) and 6 (yellow shade) out of a possible 10 represented by water [50].

Assessing the environmental impact of an analytical method depends on a number of variables including apparatus, chemicals, temperature, and sample size. Defining a method as green or non-green qualitatively is not feasible and does not differentiate between methods unless a significant change is noted. Consequently, using tools which generate quantitative results provides a convenient and more accurate way to compare and evaluate methods comprehensively. The National Environmental Methods Index was one of the earliest tools introduced and was used to evaluate hundreds of methods and made available to researchers as a free internet-searchable database [51]. Although the tool was simple and easy to use, it did not consider all aspects of the evaluated procedure and other tools were reported in the subsequent years, each with its own pros and cons [52]. To evaluate the greenness of the developed method, ESA, AGREE, and the novel ComplexMoGAPI tools were selected combining the strengths of different metrics providing a well-rounded analysis (Table 11) [53].

**Table 11:** Greenness assessment of the developed chromatographic method using the ESA, ComplexMoGAPI, and AGREE scoring techniques

Developed HPLC method	ESA	Penalty points (pp)	ComplexMoGAPI pictogram
Reagents	TEA (0.07 ml) *Pictogram (4)* *Signal word:Danger*	8	
	Tetrabutylammonium hydrogen sulphate (0.18 ml) *Pictogram (2)* *Signal word: Danger*	4	
	Phosphoric acid (< 10 ml) *Pictogram (1)* *Signal word: Danger*	2	
	Potassium phosphate *Pictogram (0)* *Signal word: Non-hazardous*	0	
	Acetonitrile (1.47 ml) *Pictogram (2)* *Signal word: Danger*	4	
Instrument	LC ≤ *1.5 KWh/sample*	1	
	Waste (15 ml)	5	
	Occupational hazard	0	
	Total pp: 24 Analytical eco scale: 76 “Excellent green analysis”		
AGREE pictogram			BAGI pictogram
	

The ESA tool ranked the developed procedure as “excellent green analysis” scoring above 75 points. Although some of the components of the mobile phase had hazardous pictograms, their use was inevitable to attain optimal peak resolution. However, minimal volumes were incorporated reducing their hazardous impact.

The ComplexMoGAPI pictogram presents other aspects of the procedure compared to ESA such as sample collection and handling and sample preparation, as well as choice of reagents and solvents, instrumentation and associated procedures [44]. The classification of the hazardous effects of reagents is based on the National Fire Protection Association (NFPA) score as opposed to hazardous pictograms in ESA. The visual representation of the results demonstrated the weaknesses of the method indicated by the red sections where off-line analysis and generation of more than 10 ml of waste (15 ml/run) were not favourable. However, over 93% of the generated waste was composed of water with a lower harmful impact on the environment. Procedures with moderate environmental impact were highlighted by the yellow sections where the use of some non-green reagents posed environmental concerns, however, their small volumes and associated NFPA scores classified them as intermediate hazards. The majority of the sections of the pictogram were shaded green reflecting green procedures where direct analysis was performed with no derivatization, safe sample handling processes with low energy and hazardous chemical consumption. An additional hexagon was included as a modification of the original GAPI tool [54] to assess “Yield and Conditions” of the procedure. Fields I, II, and VI were not shaded as they were related to product synthesis therefore, they were not shaded on the pictogram. ComplexMoGAPI proposes a quantitative score and a shaded scale for easier representation of the assessment. The developed method scored 76 with a green shade indicating good environmental sustainability, similar score to ESA method.

In addition, the AGREE tool is another metric that provides both quantitative and visual measures and considers the 12 principles of GAC. The developed method scored 0.53 out of 1 with a corresponding light green shade. Looking at the sectors of the pictogram, the weakness of the method was demonstrated by a red colour (sector 3) due to the procedure being an off-line analysis, however, the use of small sample volumes, few sample preparation steps, and no derivatizing agents were considered favourable green practices. The remaining sectors were given different shades of yellow reflecting intermediate green practices, namely the generation of water-based waste, the throughput of 4 samples per hour, and the use of liquid chromatography as opposed to a high energy-consuming apparatus.

While evaluating the greenness of a method is important to assess its environmental impact, an appropriate balance between greenness and practicality is essential. The evaluation of the method’s greenness was complemented by BAGI [46], which focuses on the method’s practicality (blueness).

The BAGI is a novel metric tool based on ten attributes including the ease of an analytical procedure, simplicity of the instrument, and availability of reagents, amongst others [46]. BAGI provides a quantitative score and a visual pictogram where darker shades of blue indicate a higher score. The developed method scored (75) reflecting its strong applicability for routine use and complementing other green metrics. Key strengths include the developed method’s quantitative ability, time efficiency, and reliance on common commercially available materials, and simplicity in terms of sample preparation and handling.

The results from all the tools provided a complementary perspective on the environmental impact of the developed method. While ESA highlighted the green merits of the method based on minimal hazardous impact, ComplexMoGAPI and AGREE offered a detailed assessment by incorporating aspects like sample preparation and handling, method throughput, device positioning, and other components of reagents’ safety such as NFPA scores. Together, with the blueness assessment these tools presented an inclusive evaluation, ensuring that all critical environmental factors as well as method feasibility were accounted for.

## Conclusion

In the present investigation, an accurate, precise, and selective HPLC method was developed for the quantification of LD and CD in bulk, oleogel, and release medium. The method could be employed for research and development to optimize long-acting injectables or quality control purposes. The run time of 15 min allows analysis of a large number of samples. Moreover, the methodology was assessed in the realms of green chemistry and sustainable chemistry, aiming to establish a more environmentally friendly and sustainable analytical process.

## Supplementary Information

Supplementary Material 1.

## Data Availability

The datasets used and/or analysed during the current study are available from the corresponding author on reasonable request.
