# Supplementary material for: Analytical method for simultaneous quantification of levodopa and carbidopa in the injectable oleogel formulation by HPLC
Source: BMC Chem. 2025 Feb 17;19(1):43. doi: 10.1186/s13065-025-01410-8 (PMC11834227; doi:10.1186/s13065-025-01410-8)
Supplement: Supplementary file 1 — Supplementary Material 1. [file 13065_2025_1410_MOESM1_ESM.docx]

**Supplementary:**

**Column used -** Luna-C18 column (250 ×4.6 mm, 5 µm), Column temp: 40 °C.

**Trial 1: Isocratic elution**

**Mobile phase: 75:25- Sodium phosphate pH 2.8: ACN, injection volume: 20 µL.**

**Runtime - 12 min, Flow rate: 1 ml/min.**


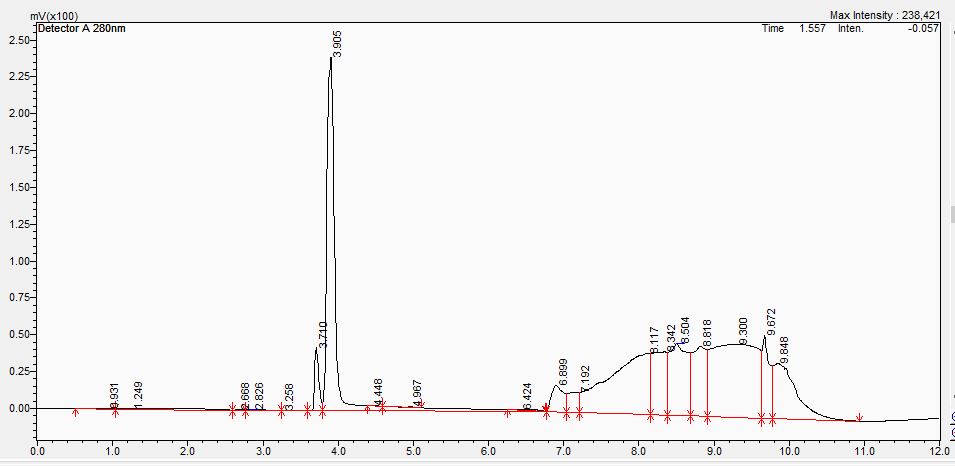


Supplementary Figure 1, Chromatogram of levodopa and carbidopa mixture

**Trial 2: Mobile phase A: Sodium phosphate pH 2.8**

**Mobile phase B: ACN, injection volume: 20 µL**

**Run time - 12 min, Flow rate :1 ml/min.**

**Gradient:**

| Time | Mobile phase A | Mobile phase B |
| --- | --- | --- |
| 0 | **90** | **10** |
| 3 | **90** | **10** |
| 5 | **10** | **90** |
| 7 | **10** | **90** |
| 9 | **90** | **10** |
| 12 | **90** | **10** |


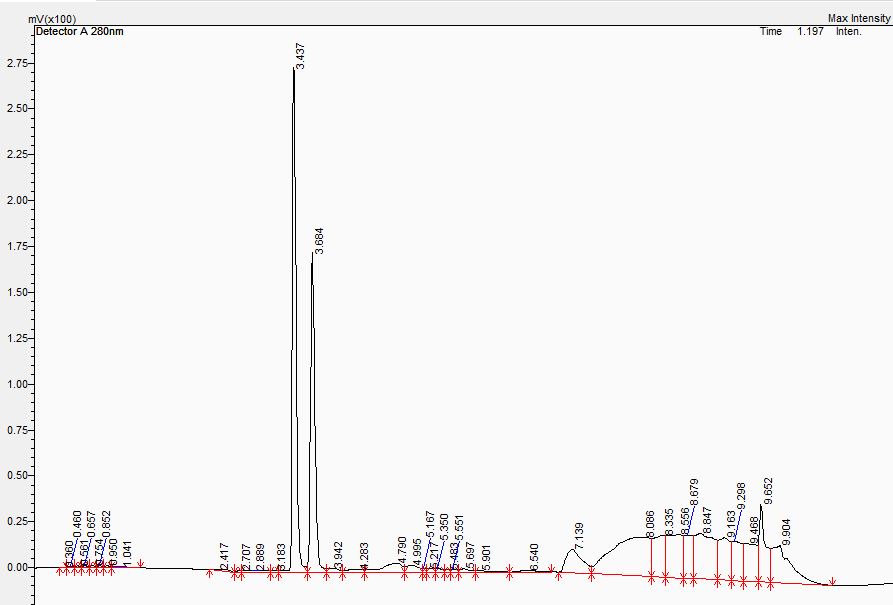
Supplementary Figure 2, Chromatogram of levodopa and carbidopa from trial 2.

**Trial 3: Mobile phase A: Potassium phosphate 10mM (pH2.5): ACN -95:5**

**Mobile phase B: Buffer: ACN- 50:50, injection volume: 20µL**

**Run time: 20min, Flow rate 1.2ml/min.**

**Gradient:**

| Time | Mobile phase A | Mobile phase B |
| --- | --- | --- |
| 0 | **100** | **0** |
| 2 | **100** | **0** |
| 10 | **0** | **100** |
| 12 | **0** | **100** |
| 14 | **100** | **0** |
| 20 | **100** | **0** |


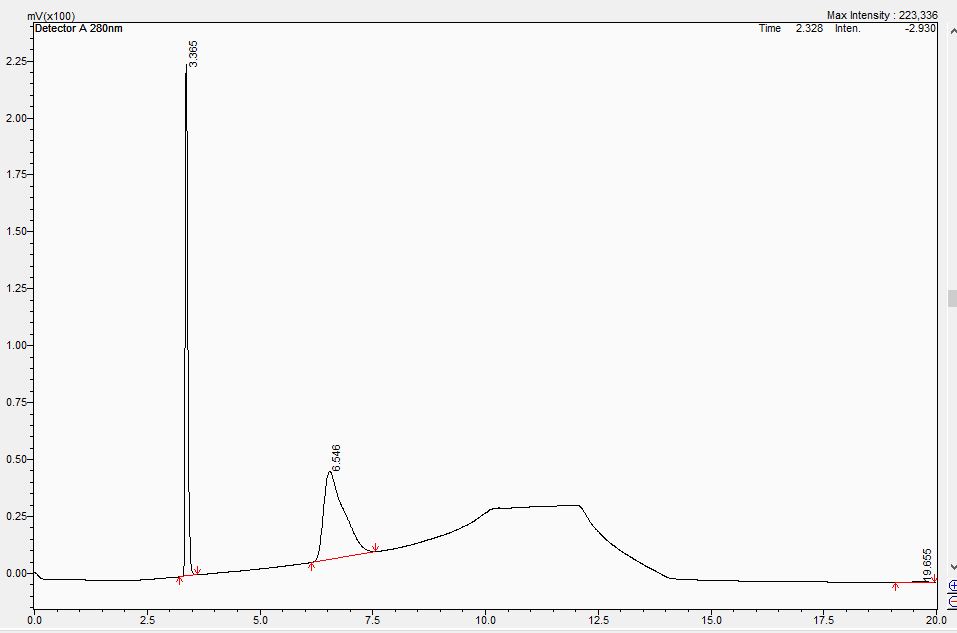


Supplementary Figure 3, Chromatogram of levodopa and carbidopa from trial 3.

Tailing was observed to avoid this we added, 0.5% Triethylamine in 10mM potassium phosphate buffer and pH was adjusted to 3.20 with orthophosphoric acid.

**Trial 4:**

**Mobile phase A: Potassium phosphate 30mM (pH 3.2): ACN -95:5**

**Mobile phase B: Buffer: ACN- 50:50, injection volume: 20 µL**

**Run time: 20 minutes, Flow rate 1.2 ml/min.**

**Gradient:**

| Time | Mobile phase A | Mobile phase B |
| --- | --- | --- |
| 0 | **100** | **0** |
| 2 | **100** | **0** |
| 10 | **0** | **100** |
| 12 | **0** | **100** |
| 14 | **100** | **0** |
| 20 | **100** | **0** |


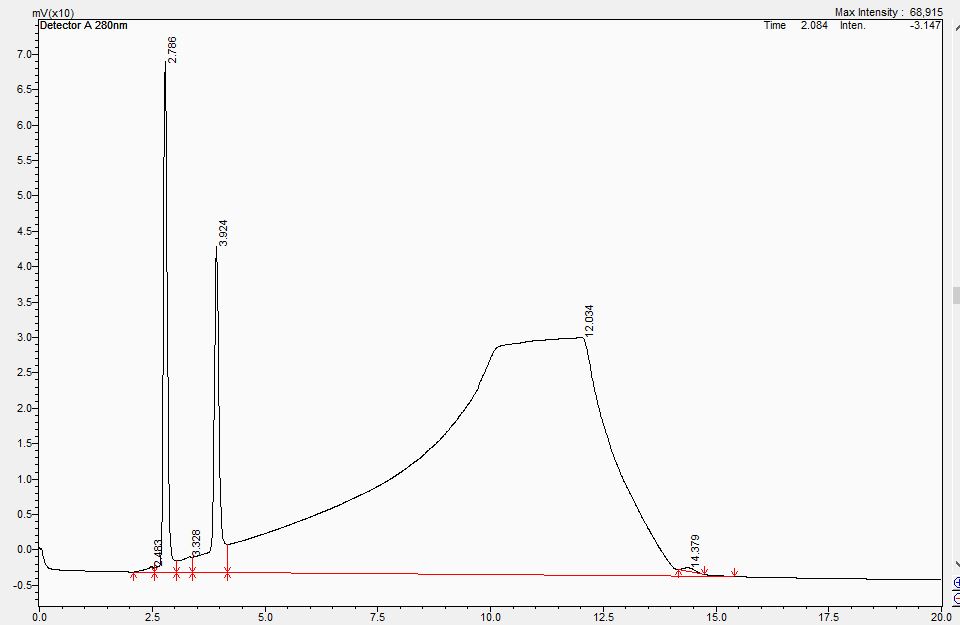


Supplementary Figure 4, Chromatogram of levodopa and carbidopa from trial 4.

Trial 4 gave Levodopa and carbidopa peaks well separated from each other.

Supplementary Figure 5, Chromatogram of levodopa from the release samples.


Supplementary Figure 6, Chromatogram of Carbidopa from the release samples.

Supplementary Figure 7, Chromatogram of Sodium bisulfite.

As shown in Figures 5 and 6, the sodium bisulfite peak begins to merge with the drug peaks, hence so achieving complete separation, Tetrabutylammonium hydrogen sulphate (TBHAS) was added at 35 mM concentration in the mobile phase A.

Supplementary Figure 8, Chromatogram of levodopa and carbidopa in the presence of sodium bisulfite. Another Trial was run to minimize the run time.

**Trial 5: Mobile phase A: Potassium phosphate 30 mM (pH 3.2): ACN -95:5**

(Containing 35 mM tetrabutylammonium hydrogen sulphate)

**Mobile phase B: Buffer: ACN- 50:50, injection volume: 20µL**

**Flow rate: 1 ml/min.**

| Time (min) | Mobile phase A | Mobile phase B |
| --- | --- | --- |
| 0 | 100 | 0 |
| 2 | 100 | 0 |
| 10 | 80 | 20 |
| 12 | 100 | 0 |
| 15 | 100 | 0 |

**Gradient:**

Supplementary figure 9, Chromatogram of levodopa and carbidopa in presence of sodium bisulfite.
